# Effects of different light conditions on transient expression and biomass in *Nicotiana benthamiana* leaves

**DOI:** 10.1515/biol-2022-0732

**Published:** 2023-10-14

**Authors:** Yuejing Zhang, Yi Ru, Zhenzhen Shi, Hanqi Wang, Ji Zhang, Jianping Wu, Hailong Pang, Hanqing Feng

**Affiliations:** College of Life Science, Northwest Normal University, Lanzhou, 730070, Gansu, China; Lanzhou Veterinary Research Institute, Chinese Academy of Agricultural Science, Lanzhou 730046, Gansu, China; New Rural Development Research Institute, Northwest Normal University, Lanzhou, 730070, Gansu, China; State Key Laboratory of Aridland Crop Science, Gansu Agricultural University, Lanzhou, 730070, Gansu, China

**Keywords:** *Nicotiana benthamiana*, transient expression, light intensity, photoperiod, biomass parameters

## Abstract

In the process of the production of recombinant proteins by using an *Agrobacterium*-mediated transient gene expression system, the effectiveness of the control of light conditions pre- and post-agroinfiltration on efficiency of transient expression is worth being evaluated. In this study, *Nicotiana benthamiana* plants were used as a bioreactor to investigate the effects of different light conditions pre- and post-agroinfiltration on the transient expression of green fluorescent protein (GFP). The results showed that the plants grown under light condition for 5 weeks had the highest level of transient expression among those grown for 4–8 weeks. In the pre-agroinfiltration, the level of transient expression of GFP was obviously decreased by the increase in light intensity or by the shortening of the photoperiod. Although the shortening of the photoperiod post-agroinfiltration also decreased the level of transient expression, moderate light intensity post-agroinfiltration was needed for higher level of transient expression efficiency. However, there was no strong correlation between the transient expression efficiency and plant growth. The results suggested that light condition was an important factor affecting the level of transient expression in plants. Hence, light conditions should be optimized to obtain higher productivity of recombinant protein from transient expression systems.

## Graphical abstract

**Figure:**
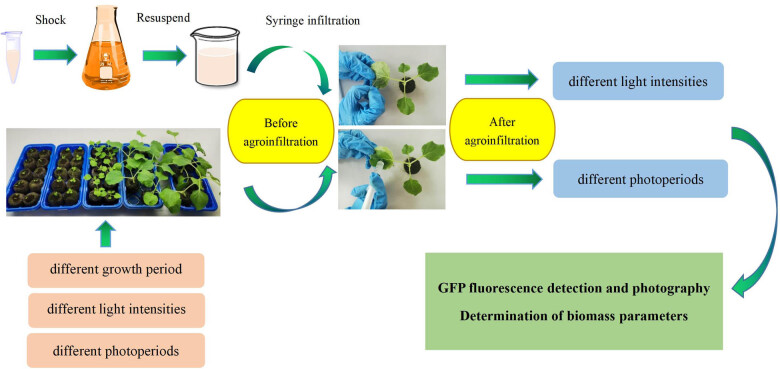

## 1 Introduction

Transgenic plants are an effective system for the study of the function, expression, and regulation of target genes. And, in recent years, transgenic plants have become the main platform for the production of recombinant pharmaceutical proteins [1,2]. The platform for the production of plant-based recombinant protein is more reliable, scalable, low-cost, and safe than the traditional expression systems using cell cultures from mammals, yeast, and bacteria [3,4]. More importantly, since plants are important food sources for human and animals, it is expected that transgenic plants can be used to prepare oral recombinant proteins or vaccines, without need of protein purification, which represents a significant advantage for plants over traditional expression systems [5–7].

Despite the obvious advantages of plants as efficient expression platforms, achieving high recombinant protein yields remains a challenge [8]. There are two major methods to express the target genes in plants. The first one is to develop a stable transgenic line, in which the gene coding the desired protein is inserted into the plant genome and its expression is derived by certain promoter, such as the cauliflower mosaic virus 35S promoter. Through succeeding generations, the foreign gene inserted in the genomes becomes heritable. The second one is to express the desired protein in plants through transient expression [2,9,10]. For the gene transient expression, the target gene is inserted in the vector and is delivered into the plant cells via *Agrobacterium tumefaciens* through a process called agroinfiltration (i.e., the vector containing the expression cassette of the gene of interest is carried by *A. tumefaciens*, which is used to infiltrate plants and thus deliver the gene of interest into the plant cells). After entering plant cells, the most of the target gene remains episomal, instead of integrating into the genome, and can be transcriptionally competent [11–14]. Compared with time-consuming stable transgenic lines, the production of desired proteins by transient expression has obvious advantages, including less time with more protein expression, consistency in protein accumulation with lower cost, and easy manipulation without any biosafety concerns. Additionally, the production of pharmaceutical proteins, antibody, or vaccine in plant cells by transient expression system would allow to rapidly cope with new diseases and mutation pathogens in our day-to-day life [15–17].

In order to improve the efficiency of transient gene expression in plants, much attention is focused on the modification of the functional region of the vector. These modification include design of 5′ and 3′ untranslated fragment, use of strong promoters, and addition of enhancer [18–20]. However, there are very few studies on whether plant growth condition and external environmental factor(s) could be utilized to improve the efficiency or increase protein yields from transient gene expression in plants.

It is well known that light is one of the most important environmental factors affecting plant physiology by deriving photosynthesis or acting as signaling. It has been reported that light intensity, photoperiod, and growth period under light condition have deep influences on the morphogenesis, development, and growth of plants, thus determining the production and the quality of plants [21,22]. However, less attention has been paid to the importance of light conditions on the efficiency of transient gene expression in plants. In fact, the plants for the transient gene expression must be grown under certain light condition, and light intensity and photoperiod can be controlled, especially when greenhouse is considered as the factory to produce valuable recombinant/therapeutic proteins in demand [23,24]. Hence, if light can actually impact the efficiency or protein yields from transient gene expression in plants, it is expected that light conditions in the process of the transient gene expression should be largely optimized.

In addition, different stages during the transient gene expression in plants would be considered carefully for the optimization of light condition. As described above, before infiltration with *A. tumefaciens*, the plants have experienced normal growth for a certain period. After infiltration with *A. tumefaciens*, an additional growth period is needed for the plants to complete the expression of the target genes and the accumulation of the desired proteins [25,26]. For achieving higher efficiency of transient gene expression or higher production of the desired proteins, one can assume that pre-agroinfiltration and post-agroinfiltration could require different light conditions. However, such issue has not been extensively investigated.

In this work, by using green fluorescent protein (GFP) as a reporter, we showed that light conditions, including growth period under light condition, light intensity, and photoperiod impact the efficiency of transient expression in *Nicotiana benthamiana* leaves during pre-agroinfiltration and post-agroinfiltration. We believe that this study would be helpful in developing new understanding on the effect of light conditions on the efficiency of transient gene expression. At the same time, this research would also be helpful in understanding how to utilize light to improve the yields of pharmaceutical protein, vaccine, and antibody, which are produced by transient gene expression in plants grown in green house with highly-controlled light condition.

## 2 Materials and methods

### 2.1 Plant material

Peat pellets were placed into a propagation tray, and water was added into the propagation tray. After the peat pellets absorbed the water for 1 h, two *N. benthamiana* seeds were added into each peat pellet and the tray was covered with a transparent plastic dome and placed at 25°C with 50% humidity. The light intensity and light: dark regime are indicated in Section 2.4, and where not indicated were applied at 100 μmol m^−2^ s^−1^ and 16/8 h. Two weeks after planting, the dome was removed, the water in the tray was drained, and Jack’s fertilizer with a concentration of 1.48 g/L was added. The plants continued to grow in the same environmental conditions as described above, and Jack’s fertilizer was supplied every day. In the third week, the plants with peat pellets were transferred to a new tray to provide adequate space for further growth until they are ready to be infiltrated at the time point needed.

### 2.2 Preparation of bacterial cultures for plant transformation

*A. tumefaciens* LBA4404 strains harboring the binary vector with the GFP gene were streaked on *Agrobacterium* rhizogene medium (0.5% peptone, 0.1% yeast extract, 0.5% beef extract, 0.5% sucrose, 0.05% MgSO_4_, 1.5% agar, pH 7.4) containing kanamycin (50 μg/mL), rifampicin (25 μg/mL), and chloramphenicol (25 μg/mL). Similarly, the EHA105 strains harboring the same vector were streaked on *Agrobacterium* rhizogene medium containing kanamycin (50 μg/mL) and rifampicin (25 μg/mL). After 24 h growth, the monoclone were picked out from the *Agrobacterium* rhizogene medium and inoculated into 50 mL *Agrobacterium* rhizogene broth medium (0.5% peptone, 0.1% yeast extract, 0.5% beef extract, 0.5% sucrose, 0.002% MgSO_4_, pH 7.4) with antibiotics, at 28°C overnight in a shaker at 180 rpm. The bacteria were collected by centrifugation at 5,000 rpm for 10 min and then washed by infiltration buffer (10 mM MES-KOH, pH 5.5; 10 mM MgSO_4_, 100 μM acetosyringone). Then, the bacteria were collected again and re-suspended in the infiltration buffer. The bacterial concentrations were determined by measuring optical density (OD) at 600 nm and were diluted to the concentrations needed.

### 2.3 Syringe infiltration

*N. benthamiana* leaves with the same size were selected, and 2 mL of bacterial suspension was taken up with a 5 mL sterile syringe. Then a small nick was created with a needle in the epidermis on the back side of the leaf. The *A. tumefaciens* in infiltration buffer was injected into the nick with a syringe without a needle.

### 2.4 Material treatments

In the first set of the experiment, the 4-, 5-, 6-, 7-, or 8-week-old *N. benthamiana* plants that were grown under the conditions as described in Section 2.1 were used for the infection with LBA4404 or EHA105 strain. For LBA4404 strain infection, the *A. tumefaciens* concentration was set at OD at a wavelength of 600 nm (OD_600_) 0.8, and the infected plants were incubated for 2 days under the conditions as described in Section 2.1. For EHA105 strain infection, the *A. tumefaciens* concentration was set at OD_600_ 0.6, and the infected plants were incubated for 4 days under the conditions as described in Section 2.1.

In the second set of the experiment, the 5-week-old *N. benthamiana* plants that were grown under different light intensities at 50, 100, 150, 200, and 250 μmol m^−2^ s^−1^, respectively, were used for the infection with LBA4404 or EHA105 strain. The *A. tumefaciens* concentration and the time of incubation was the same as those in the first set of the experiment.

In the third set of the experiment, the 5-week-old *N. benthamiana* plants, which were grown under different light: dark regime at 16/8, 12/12, and 8/16 h, respectively, were used for the infection with LBA4404 or EHA105 strain. The *A. tumefaciens* concentration and the time of incubation was the same as those in the first set of the experiment.

In the fourth set of the experiment, the 5-week-old *N. benthamiana* plants were used for the infection with LBA4404 or EHA105 strain. The *A. tumefaciens* concentration was the same as those in the first set of the experiment. After the LBA4404 strain infection, the infected plants were incubated for 2 days under different light intensities at 50, 100, 150, 200, and 250 μmol m^−2^ s^−1^, respectively. After the EHA105 strain infection, the infected plants were incubated for 4 days under different light intensities at 50, 100, 150, 200, and 250 μmol m^−2^ s^−1^, respectively.

In the fifth set of the experiment, the 5-week-old *N. benthamiana* plants were used for the infection with LBA4404 or EHA105 strain. The *A. tumefaciens* concentration was the same as those in the first set of the experiment. After the LBA4404 strain infection, the infected plants were incubated for 2 days under different light: dark regime at 16/8, 12/12, and 8/16 h, respectively. After the EHA105 strain infection, the infected plants were incubated for 4 days under different light: dark regime at 16/8, 12/12, and 8/16 h, respectively.

The concentration of bacterial solution and infiltration days of above experiments were the best conditions for GFP expression, which were obtained from the screening of the previous experiments.

### 2.5 GFP fluorescence detection and photography

The fluorescence from the GFP in the infected leaves was detected and photographed by fluorescence stereoscope (Leica M205 FA, Germany) with excitation wavelength of 450–490 nm and emission wavelength of 500–550 nm. Fluorescence images were quantitatively analyzed with Image J software.

### 2.6 Biomass parameters

The leaves of the plants were taken and photographed, and the area of the leaves was measured by Photoshop software. The fresh weight was immediately measured and recorded using an analytical balance. The fresh samples were dried at 70°C in an oven, until a constant weight was obtained, and the dry weight was measured immediately. All the above biomass parameters were measured immediately after GFP fluorescence detection.

### 2.7 Statistical analysis

The value obtained was the average value of at least three independent experiments, and the data were mean values of at least three plants. Data were analyzed via one-way analysis of variance with SPSS statistical software (Version 20.0, IBM Corporation). The data were analyzed with Duncan’s multiple comparison. A probability level of 0.05 is considered as statistical significance. Pearson correlation coefficients were calculated using SPSS software (version 20.0, IBM Corporation).

## 3 Results

### 3.1 Effects of growth period under light condition on transient expression and biomass parameters

**Figure 1:**
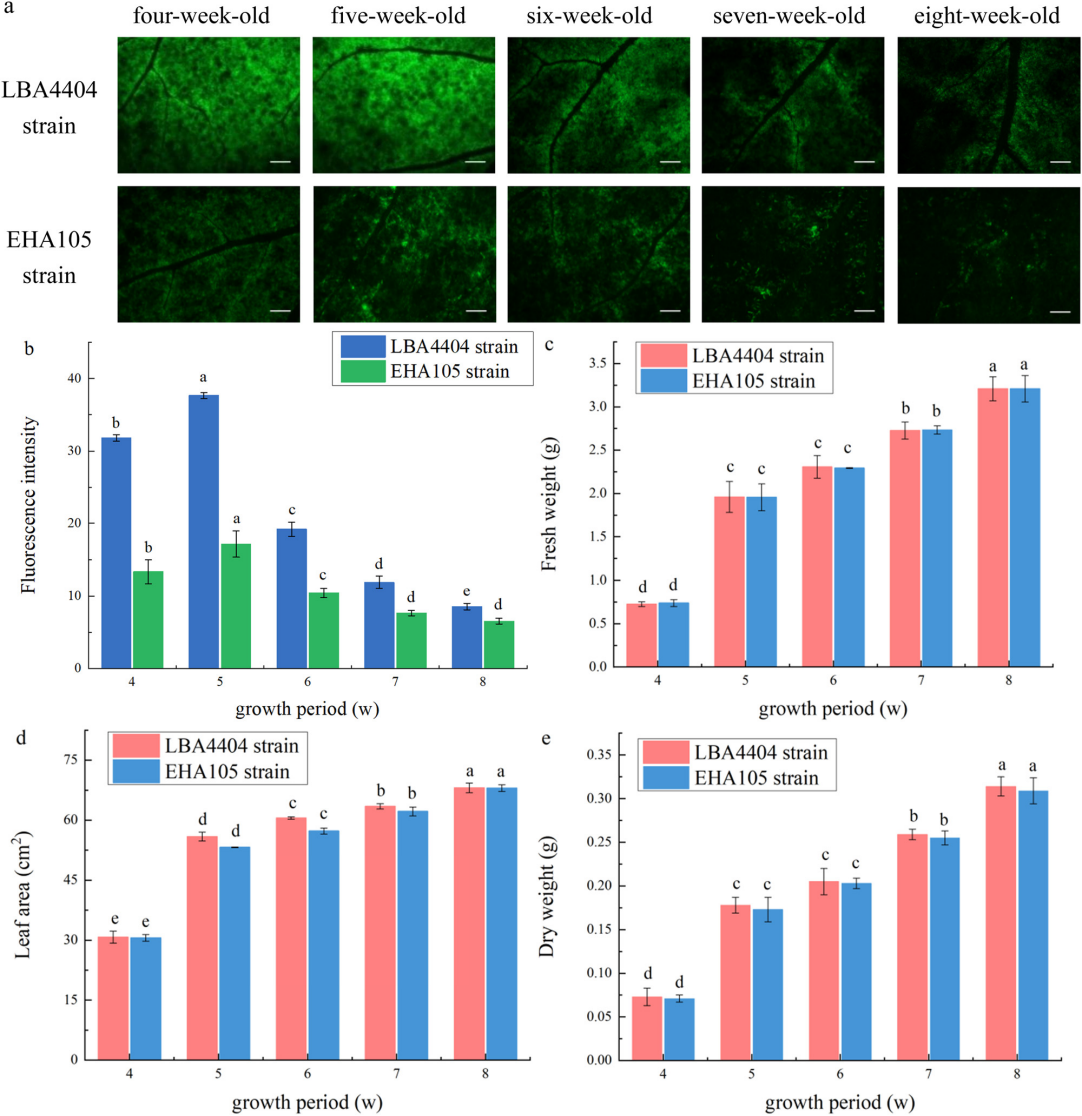
Changes of fluorescence intensity and biomass parameters of leaves infected with LBA4404 and EHA105 strains at different ages. (a) Representative images indicating the green fluorescence intensity of plants infected with LBA4404 and EHA105 strains at 4-, 5-, 6-, 7-, or 8-week-old, Bar = 2 mm. (b) Green fluorescence intensity of plants infected with LBA4404 and EHA105 strains at 4-, 5-, 6-, 7-, or 8-week-old. (c) Fresh weight, FW. (d) Leaf area, LA. (e) Dry weight, DW. Different lowercase letters indicate significant differences (*P* < 0.05) among the different treatments.

The growth period under light condition (i.e., the age of plants) could be one of the important factors that affect the yield of recombinant protein. We first studied the effects of growth period under light condition on transient expression of GFP gene, which were produced by the infection of LBA4404 and EHA105 strains. Compared with the control groups (LBA4404 and EHA105 strains without GFP), infection with LBA4404 or EHA105 strain carrying GFP gene induced transient expression of GFP (Figure S1c and Figure 1). We compared the green fluorescence from GFP expression among the plants with different growth period under light condition. The result showed that, with the increase of growth period under light condition (from 4 to 8 weeks), the green fluorescence from GFP expression increased first and then decreased. As a whole, the fluorescence from GFP expression in the plants infected with LBA4404 strain was higher than those infected with EHA105 strain. The 5-week-old plants had the highest level of fluorescence among all the plants with different growth period (Figure 1a and b). Thus, in the work later, the 5-week-old plants were chosen as the representatives to study the effects of other light condition on transient expression.

As presented in Figure 1c–e, we also measured the biomass parameters of the plants with different growth periods under light condition, including fresh weight, dry weight, and leaf area. It was found that all of these three parameters increased with the increase of the growth period of the plants under light condition, although, there was no significant difference in fresh weight and dry weight between the 5- and 6-week-old plants. Combined the results presented in Figure 1, it is suggested that the growth period of the plants under light condition can affect the transient expression efficiency, but there was no strong negative correlation between the transient expression efficiency and biomass among the plants with different growth period under light condition (Figure S1a and b).

### 3.2 Effects of different light intensities before agroinfiltration on transient expression and biomass parameters

**Figure 2:**
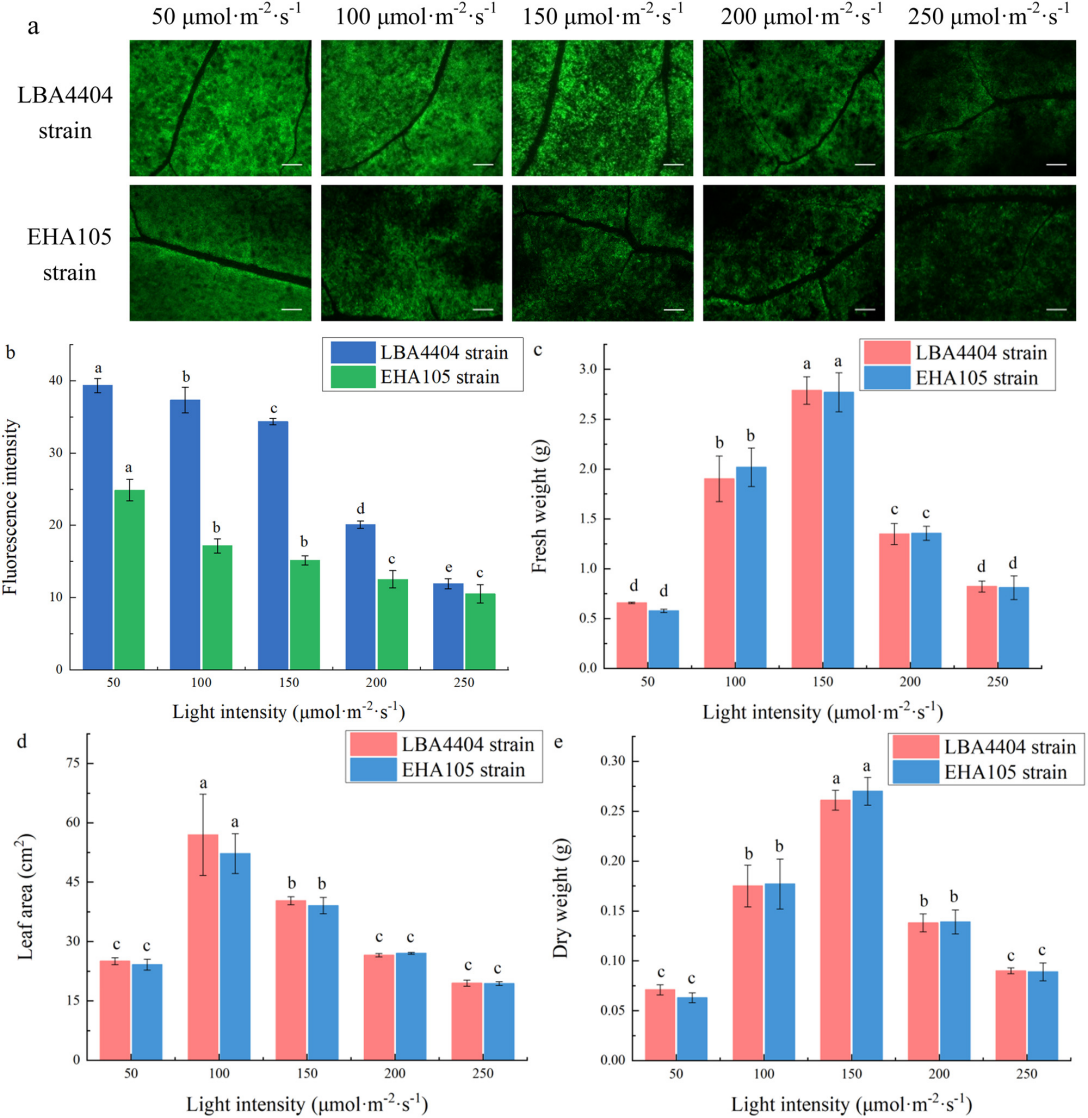
Changes of fluorescence intensity and biomass parameters of the plant leaves at different light intensities before agroinfiltration. (a) Representative pictures indicating the green fluorescence intensity of plants that were treated under the light intensities at 50, 100, 150, 200, 250 μmol m^−2^ s^−1^ before LBA4404 and EHA105 strains agroinfiltration. Bar = 2 mm. (b) Fluorescence intensity of plants under the light intensities at 50, 100, 150, 200, 250 μmol m^−2^ s^−1^ before LBA4404 and EHA105 strains agroinfiltration. These plants were placed back into the original light intensities after agroinfiltration to continue incubation. (c) Fresh weight, FW. (d) Leaf area, LA. (e) Dry weight, DW. Different lowercase letters indicate significant differences (*P* < 0.05) among the different treatments.

As shown in Figure 2a and b, we studied the effects of different light intensity before agroinfiltration on transient expression of GFP gene after infection by LBA4404 and EHA105 strains. When the light intensity is at 50 μmol m^−2^ s^−1^, the fluorescence from GFP expression in the plants infected with LBA4404 and EHA105 strains was the highest among the other light intensities. With the increase of light intensity from 50 to 250 μmol m^−2^ s^−1^, the green fluorescence from GFP expression decreased. Additionally, the measurement of the biomass parameters showed that with the increase of the light intensity (from 50 to 250 μmol m^−2^ s^−1^) before agroinfiltration, the fresh weight, dry weight, and leaf area of the plant increased first but then decreased. The fresh and dry weight peaked at 150 μmol m^−2^ s^−1^, while the leaf area peaked at 100 μmol m^−2^ s^−1^ (Figure 2c–e). These results showed that the light intensity before agroinfiltration can affect the GFP expression, and moderate light intensity can enhance plant growth while excessive light intensity would inhibit the growth of plants. Combined the results presented in Figure 2, it is suggested that there was no strong correlation between the transient expression efficiency and biomass parameters among the plants with different intensity before agroinfiltration (Figure S1a and b).

### 3.3 Effects of different light/dark regime before agroinfiltration on transient expression and biomass parameters

**Figure 3:**
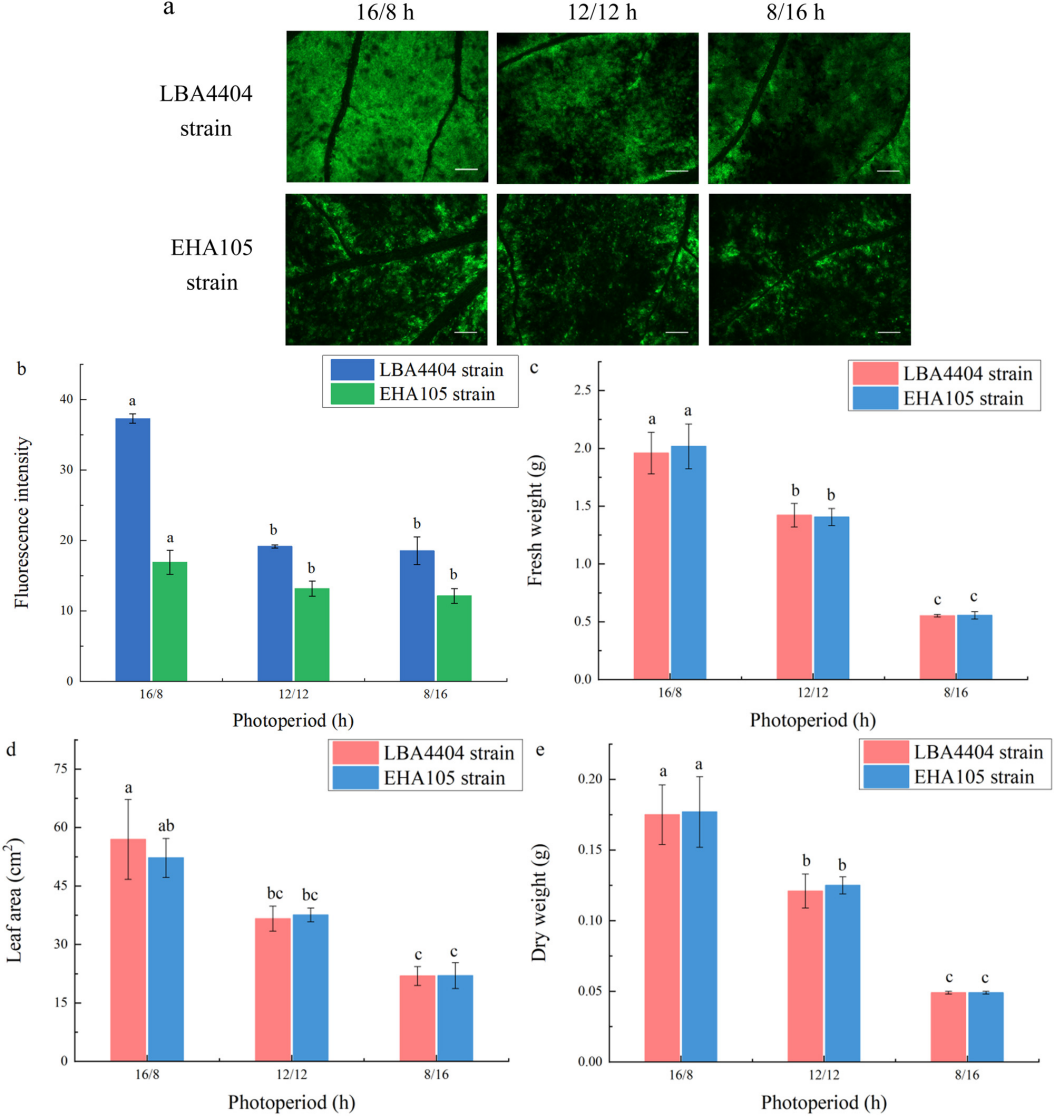
Changes of fluorescence intensity and biomass parameters of the plant leaves at different photoperiods before agroinfiltration. (a) Representative pictures indicating the green fluorescence intensity of plants that were treated under the photoperiods at 16/8, 12/12, and 8/16 h before LBA4404 and EHA105 strains agroinfiltration. Bar = 2 mm. (b) Fluorescence intensity of plants under the photoperiods at 16/8, 12/12, and 8/16 h before LBA4404 and EHA105 strains agroinfiltration. These plants were placed back into the original photoperiods after agroinfiltration to continue incubation. (c) Fresh weight, FW. (d) Leaf area, LA. (e) Dry weight, DW. Different lowercase letters indicate significant differences (*P* < 0.05) among the different treatments.

The fluorescence from GFP expression was highest when the light/dark regime before agroinfiltration was 16/8 h among the plants with three different light: dark regimes (16/8, 12/12, or 8/16 h). Decrease in photoperiod from 16/8 to 12/12 h reduced fluorescence from GFP expression. However, there was no significant difference in fluorescence from GFP expression between the light: dark regime at 12/12 h and at 8/16 h (Figure 3b).

With the shortening of photoperiod before agroinfiltration, leaf area decreased. The changes in the values of fresh weight and dry weight under different light/dark regime before agroinfiltration presented a similar pattern with the changes of leaf area (Figure 3c–e). This indicates that the light: dark regime at 16/8 h before agroinfiltration was more beneficial to the growth of plants. Combined the results presented in Figure 3, it is suggested that there was no strong positive correlation between the transient expression efficiency and biomass parameters among the plants grown with different light/dark regime before agroinfiltration (Figure S1a and b).

### 3.4 Effects of different light intensities after agroinfiltration on transient expression and biomass parameters

**Figure 4:**
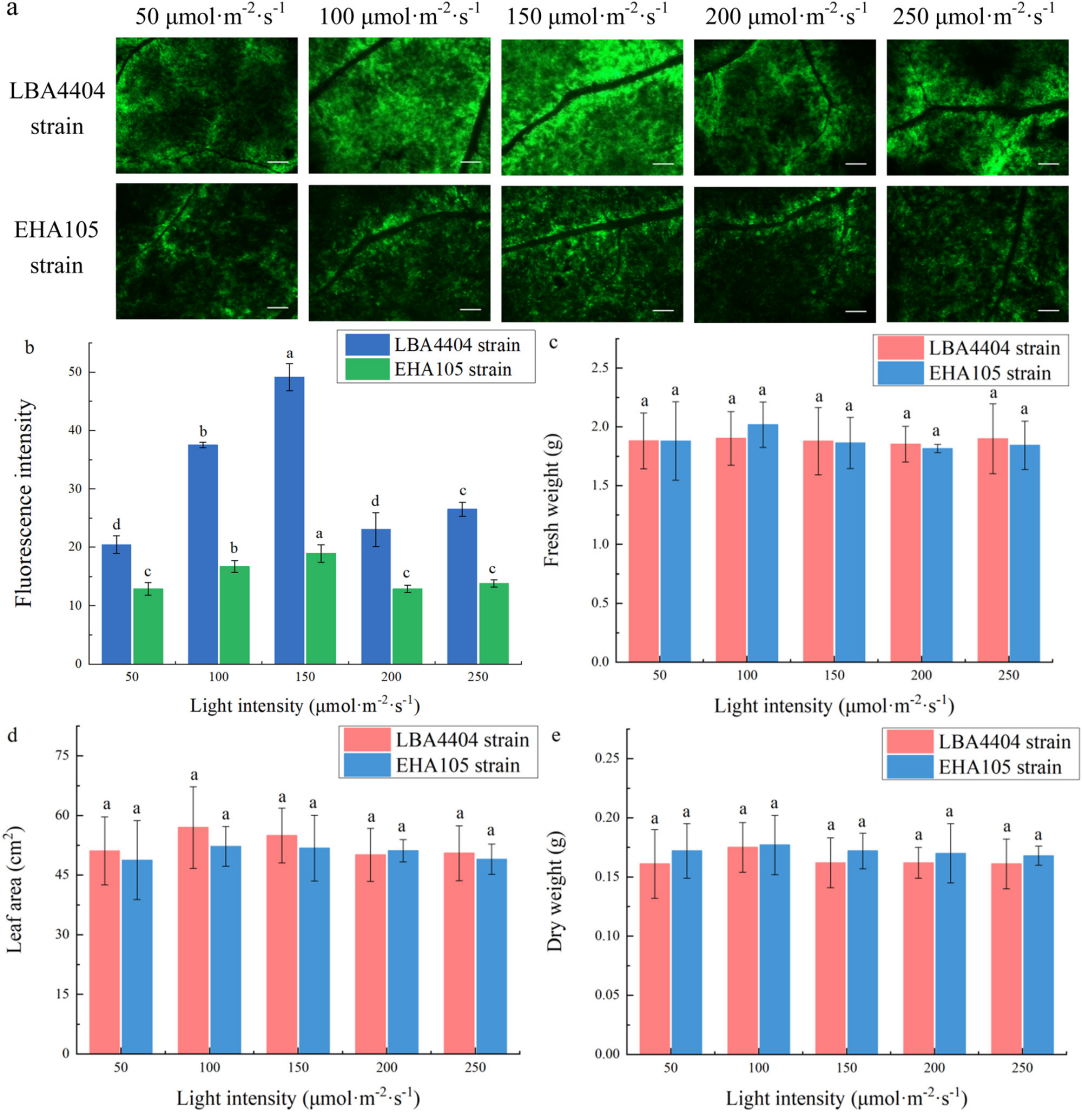
Changes of fluorescence intensity and biomass parameters of the plant leaves at different light intensities after agroinfiltration. (a) Representative pictures indicating the green fluorescence intensity of plants that were treated under the light intensities at 50, 100, 150, 200, 250 μmol m^−2^ s^−1^ after LBA4404 and EHA105 strains agroinfiltration. Bar = 2 mm. (b) Fluorescence intensity of plants under the light intensities at 50, 100, 150, 200, 250 μmol m^−2^ s^−1^ after LBA4404 and EHA105 strains agroinfiltration. (c) Fresh weight, FW. (d) Leaf area, LA. (e) Dry weight, DW. Different lowercase letters indicate significant differences (*P* < 0.05) among the different treatments.

Figure 4 shows the effects of different light intensities after agroinfiltration on the green fluorescence from GFP expression. The results indicated that with the increase of light intensity (from 50 to 250 μmol m^−2^ s^−1^) after agroinfiltration, the fluorescence from GFP expression increased first and then decreased. The fluorescence from GFP expression peaked at 150 μmol m^−2^ s^−1^, which was obviously higher than that at other light intensities after agroinfiltration.

There was no significant difference in the fresh weight of the infected plants at different light intensities after agroinfiltration. Similarly, leaf area and dry weight did not change significantly at different light intensities after agroinfiltration (Figure 4c–e). The results showed that the light intensity after agroinfiltration had no effect on the fresh weight, dry weight, and leaf area. There was no correlation between the transient expression efficiency and biomass parameters among the plants at different light intensities after agroinfiltration.

### 3.5 Effects of different light/dark regime after agroinfiltration on transient expression and biomass parameters

**Figure 5:**
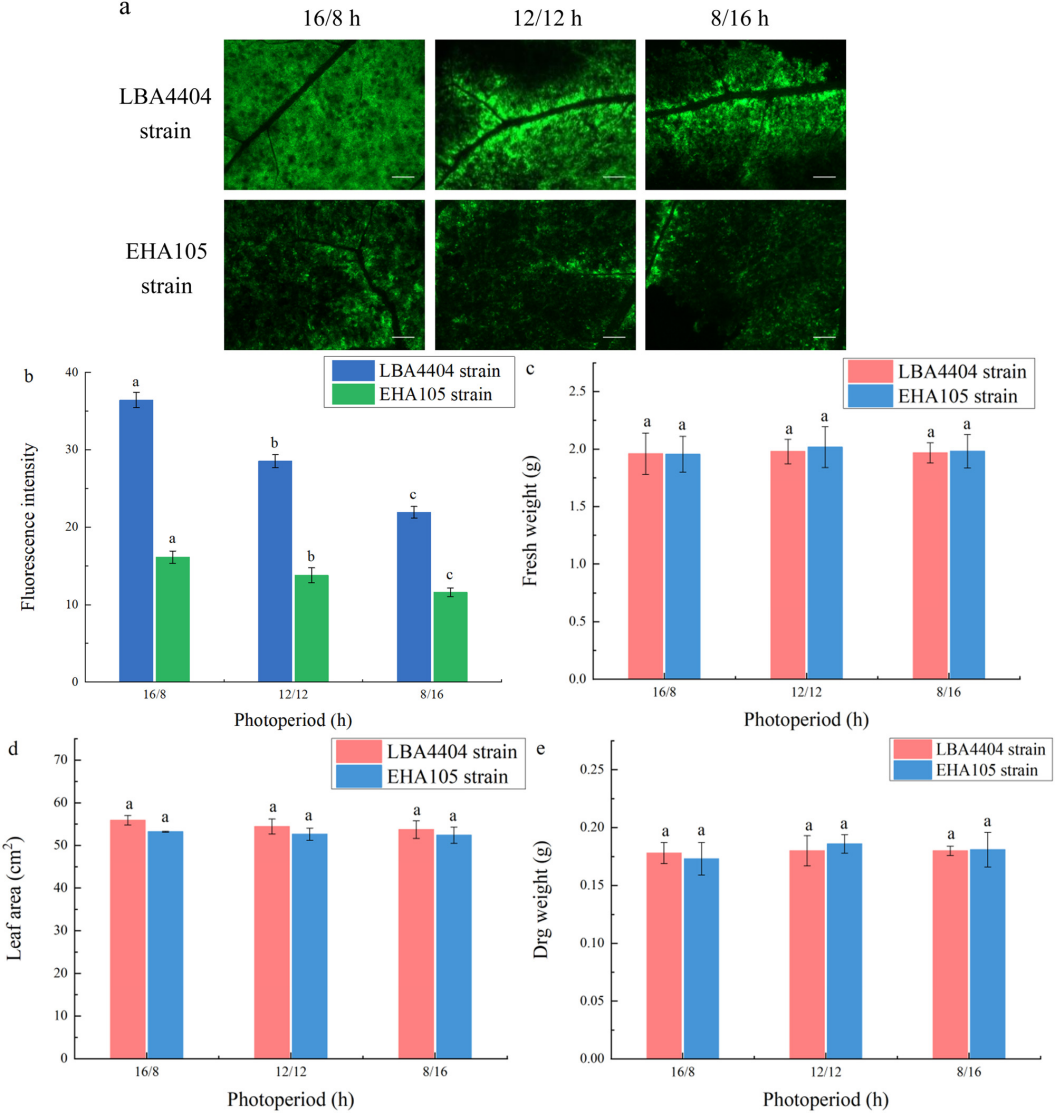
Changes of fluorescence intensity and biomass parameters of the plant leaves at different photoperiods after agroinfiltration. (a) Representative pictures indicating the green fluorescence intensity of plants that were treated under the photoperiods at 16/8, 12/12, and 8/16 h after LBA4404 and EHA105 strains agroinfiltration. Bar = 2 mm. (b) Fluorescence intensity of plants under the photoperiods at 16/8, 12/12, and 8/16 h after LBA4404 and EHA105 strains agroinfiltration. (c) Fresh weight, FW. (d) Leaf area, LA. (e) Dry weight, DW. Different lowercase letters indicate significant differences (*P* < 0.05) among the different treatments.

The fluorescence from GFP expression peaked when the light/dark regime after agroinfiltration was at 16/8 h, which was significantly higher than that at 12/12 or 8/16 h (Figure 5a and b).

No significant changes in the fresh weight, dry weight, and leaf area were observed under the different photoperiods after agroinfiltration (Figure 5). This indicates that the accumulation of plant biomass was unaffected by the different photoperiods after agroinfiltration. There was no correlation between the transient expression efficiency and biomass parameters among the plants with different light/dark regime after agroinfiltration.

## 4 Discussion

The present work mainly studied the influence of light condition on plant transient expression. The growth period under light condition (i.e., the age of plants) could be the most easily controlled light factor during plant growth. As shown in the present work, the biomass of the plants used for the transient expression increased with the increase of the age of plants (from 4 to 8 weeks) (Figure 1). However, the increase in the growth period under light condition did not correspondingly enhance the transient expression efficiency. The 5-week-old plants had highest level of fluorescence among the 4-, 5-, 6-, 7-, or 8-week-old plants (Table 1). This indicated that the production of recombinant protein requires optimal plant growth period. The transient protein expression level decreased as the plant growth period generated more biomass, reflecting the greater protein synthesis potential of young plants. Consistent with our observations, the works from group of Dodds et al. and Saur et al. found that the success of transient gene expression decreased with the age of the *N. benthamiana* plants, and they suggested that this could be attributed to the cold shock protein of *A. tumefaciens*, which can trigger immune responses including a burst of reactive oxygen species, from old plants, but not from young plants [27,28]. On the other hand, in theory, the cell walls of younger plants are more relaxed for faster cell division, compared to those in the older plants [29,30]. Previous work reported that relaxation of cell wall may render the plant more vulnerable to biotic intruders by facilitating pathogen entry, allowing enhanced nutrient leakage, and increasing availability of resources for pathogens [31]. Thus, we assume that the younger plants are most susceptible to the infection and spread of *A. tumefaciens* than the older ones and, as the result, the transient expression of foreign gene by agroinfiltration increased in the younger plants.

**Table 1:** Best conditions for fluorescence intensity and biomass parameters of leaves infected by LBA4404 and EHA105 strains under different conditions

*Agrobacterium* strains	Fluorescence intensity and biomass parameters	Growth period (w)	Before agroinfiltration		After agroinfiltration	
			Light intensity (μmol m^−2^ s^−1^)	Photoperiod (h)	Light intensity (μmol m^−2^ s^−1^)	Photoperiod (h)
LBA4404	Fluorescence intensity	5	50	16/8	150	16/8
	Fresh weight	8	150	16/8	—	16/8
	Leaf area	8	100	16/8	—	16/8
	Dry weight	8	150	16/8	—	16/8
EHA105	Fluorescence intensity	5	50	16/8	150	16/8
	Fresh weight	8	150	16/8	—	16/8
	Leaf area	8	100	16/8	—	16/8
	Dry weight	8	150	16/8	—	16/8

Note: “−” represents no difference in biomass parameters at different light intensities.

It is well known that light intensity and photoperiod can affect plant growth [32]. However, light conditions where the transient expression takes place in plants should also be controlled for recombinant protein accumulation, rather than only for plant growth. The light conditions for recombinant protein yield are likely different between pre- and post-agroinfiltration processes, since different biological events occur during pre- and post-inoculation [26,33]. Before agroinfiltration, light condition would make the plant more susceptible to *Agrobacterium.* In contrast, after agroinfiltration, light control would aim to enhance the transient expression of recombinant protein. Thus, in the present work, we investigated the effect of light intensity and photoperiod in the pre- and post-agroinfiltration on the transient expression and plant growth. For *N. benthamiana* plants, increase in the light intensity (from 50 to 250 μmol m^−2^ s^−1^) before agroinfiltration decreased the level of transient expression of GFP gene (Figure 2), although light intensity before agroinfiltration from 100 to 200 μmol m^−2^ s^−1^ can enhance the plant growth (Table 1). The mechanism for the decrease of the transient expression by the increase in the light intensity before agroinfiltration is unclear. One of the possible reasons is that the increase in the light intensity before agroinfiltration could increase the resistance of the plants to *A. tumefaciens*, since previous works have reported that plants acclimated to high light displayed increased resistance against virulent *Pseudomonas syringae* pv. *tomato* DC3000 [34,35]. We also found that the shortening of the photoperiod before agroinfiltration not only decreased the plant growth but also reduced the transient expression efficiency. Recent work showed that growth under long day compared with growth under short day enhances the expression of defense-related genes and resistance of plant to the necrotrophic pathogen [36]. Thus, it is possible that light intensity and photoperiod in the pre-agroinfiltration could affect the transient expression efficiency via affecting the resistance of plant to *A. tumefaciens*, although the actual mechanism is still unknown and could be different from the assumed.

Further study in the present works showed that the effects of light intensity and photoperiod in the post-agroinfiltration on the plant growth and transient expression efficiency are obviously different from those in the pre-agroinfiltration. Although the shortening of the photoperiod after agroinfiltration, similarly to the shortening of the photoperiod before agroinfiltration, also decreased the level of transient expression (Figures 3 and 5), the increase in the light intensity after agroinfiltration did not cause the gradual decrease in the level of transient expression efficiency. Instead, the light intensity after agroinfiltration from 100 to 200 μmol m^−2^ s^−1^ effectively enhanced the level of transient expression efficiency (Table 1). In addition, the changes of the light intensity and photoperiod in the post-agroinfiltration did not obviously affect the plant growth (Figures 4 and 5). It is difficult to explain why the effects of pre- and post-inoculation light condition on the level of transient expression and plant growth are so different. On the one hand, the *A. tumefaciens*-mediated transient expression is a process involving a series of complex biological events, including *A. tumefaciens* infection, T-DNA transfer from *A. tumefaciens*, protein biosynthesis, and its accumulation in leaf tissue. Such process could inevitably affect the plant growth under light condition, possibly via affecting photosynthesis, respiration, and other metabolism [37–39]. On the other hand, the effects of light condition on plants could be more complex. Besides the effects of light on plant resistance and growth, it has been reported that light intensity and photoperiod can influence post-translational protein modifications and degradation, and other physiological process that could be related to the biological events of *Agrobacterium*-mediated transient expression [24,40–44]. For example, Zambre et al. observed that light can affect the ability to T-DNA transfer possibly by changing some physiological and metabolic processes in plant cells [24]. In addition, the studies from Zhang and Zhou showed that light intensity can affect protein synthesis by regulating the synthesis and signal transduction of endogenous hormones in plants [45]. Thus, complexity would be further increased, when one would attempt to explain the reasons for the effects of pre- and post-inoculation light condition on the level of transient expression and plant growth and the reasons for the difference between both of them.

However, the aim of the present work is to present whether the light condition can affect the transient expression level in plant and how to utilize the information obtained to optimize the pre- and post-inoculation light condition for efficient recombinant protein production in *Agrobacterium*-mediated transient expression systems. This could be important for the production of plant-based recombinant proteins in the plant factories, in which illumination condition is highly controlled. Optimized illumination can largely decrease the gratuitous consumption of energy and increase the yield of recombinant proteins, compared to the unoptimized light condition. It is also noted that, besides light, air temperature and relative humidity can also affect the transient expression reactions, which involve T-DNA transfer, recombinant protein accumulation, plant defense responses, and protein degradation [46–48]. The different pharmaceutical proteins produced from different plant species could need different environmental condition. Thus, enhancement of the production of certain recombinant protein by environmental control is still a challenge. Nonetheless, compared to temperature and relative humidity, light condition is more easily to be controlled. Thus, light condition would be considered as the primary environmental factor to be applied in recombinant protein production by plant-based transient expression systems.

## 5 Conclusions

In conclusion, the present work suggests that light condition is an important factor in the process of transient expression. The effect of different light intensities and photoperiod on the expression of foreign genes is different. The 5-week-old plants had the highest level of transient expression among those grown for 4–8 weeks. In the pre-agroinfiltration, the increase in light intensity or shortening of the photoperiod can reduce the level of transient expression of GFP was obviously decreased. In the post-agroinfiltration, the shortening of the photoperiod post-agroinfiltration also decreased the level of transient expression, while moderate light intensity enhanced the level of transient expression efficiency. Totally, there was no strong correlation between the transient expression efficiency and plant growth under different light conditions. Hence, light conditions should be optimized to obtain higher productivity of recombinant protein from transient expression systems.

## Supplementary Material

Supplementary Figure
