# Supplementary material for: Effects of different light conditions on transient expression and biomass in Nicotiana benthamiana leaves
Source: Open Life Sci. 2023 Oct 14;18(1):20220732. doi: 10.1515/biol-2022-0732 (PMC10579877; doi:10.1515/biol-2022-0732)
Supplement: Supplementary Figure [file biol-2022-0732-sm.pdf]

Supplementary

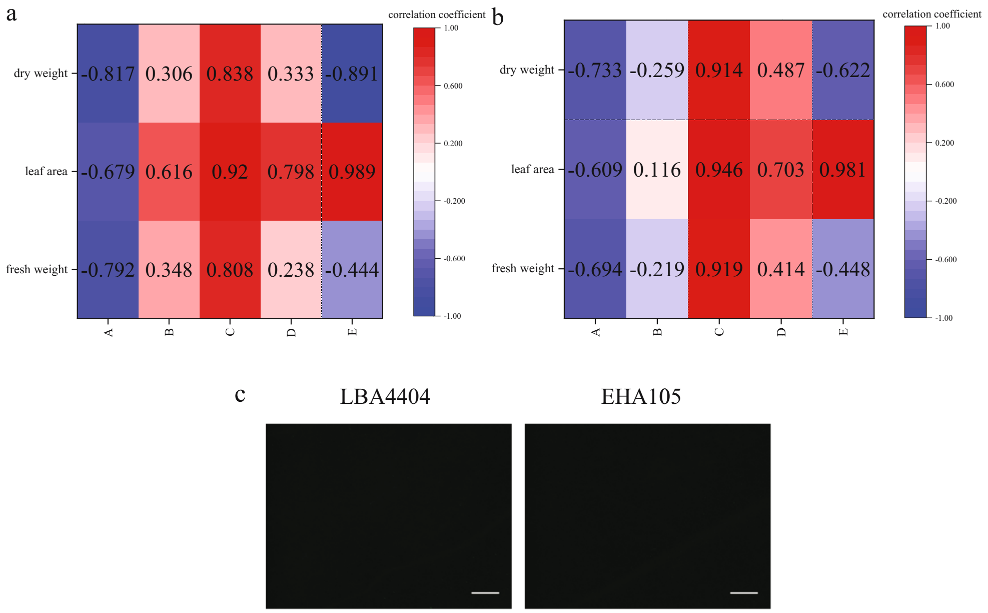

Figure S1: Correlation analysis of the green fluorescence intensity and biomass parameters under different light conditions. (a) The fluorescence intensity in the plants infected with LBA4404 strain. (b) The fluorescence intensity in the plants infected with EHA105 strain. (c) The control group of LBA4404 and EHA105 strains (LBA4404 and EHA105 strains without GFP). Note: A: different plants ages; B: different light intensity before agroinfiltration; C: different photoperiod before agroinfiltration; D: different light intensity after agroinfiltration; E: different photoperiod after agroinfiltration. * The level of significance is indicated as follows: $P < 0.05$. The data in the figure represent Pearson correlation coefficients, but there are no statistically significant correaltions.
